# Clinical Review, and Hospital Reports

**Published:** 1840-04-01

**Authors:** 


### Clinical Review.

---

### Twenty-Fifth and Twenty-sixth Annual Reports of the Directors of the Glasgow Royal Asylum for Lunatics, 1839, 1840.

We hail the appearance of these Reports from various Lunatic Asyla with feelings of unmitigated satisfaction. They are at once the evidence of improvement effected in the management of the insane, and the guarantees of continued progress in the same direction. We shall draw attention to them upon fitting opportunities, and notice whatever seems to merit it.

1. In the first of the Reports before us, (the Twenty-fifth) we are informed that, of the patients whose treatment has, in the course of the year, been concluded by dismission or death, the cured amount to 47 per cent., the dismissed uncured to 38 per cent., and the dead to 14 per cent.

Several of the cures were highly satisfactory. For example:—one of the female paupers had been for seven years a patient in the Asylum. She had, in the course of that time, been several years subject to paroxysms of fury, and afterwards (in place of these paroxysms) to severe attacks of tic doloureux; but in the course of the last six months of her treatment, she recovered completely from all her ailments, both of body and mind.

To be able, say the Reporters, to arrest attention by any motive, or any impression sufficient to counteract delusion, is often the first symptom of convalescence, whether we choose to consider it as a cause or as a consequence; and in several cases it was not a little interesting to observe the means whereby a patient sometimes seemed to be aroused from his insane dream. In one case, in which a female patient had taken the most violent and groundless dislike to her family, the tidings of the death of a son in a foreign country, excited in her the desire to see her surviving children. This desire recalled all her parental affection, instantly sweeping from her mind her insane antipathies, and restoring her at once to right feeling and to reason.

*Suicide.*—The propensity to suicide existed in no fewer than 29 cases, *viz.* 12 males, and 17 females, all of them requiring no small degree of vigilance. The attempt at starvation is often persevered in with the greatest obstinacy, and compulsory feeding was resorted to in seven cases. It was remarkable that no fewer than six of these seven patients were females, probably because the principle of imitation operates more powerfully with them than with males; and whensoever the feeding apparatus is in any case employed, the use and object of it are unavoidably known throughout the ward to all the other patients who are capable of any degree of rational reflection. In one case the operation had to be performed twice daily, and sometimes oftener (for the exhibition of wine and medicine, as well as of food), during the long period of six weeks; in others, from a few days to two or three weeks. Some were speedily convinced of the folly of resistance, and resumed, in the course of a day or two, the natural use of their powers of swallowing.

*Mortality.*—The number of patients who died, and the denomination of the diseases which proved fatal, were as follows, viz.—

| | *Males.* | *Females.* | *Total.* |
|---|---|---|---|
| Of Abscess .. .. .. .. .. | 2 | 0 | 2 |
| Apoplexy .. .. .. .. | 2 | 1 | 3 |

| | Males. | Females. | Total. |
|---|---|---|---|
| Bruises and Sores .. .. .. | 1 | 1 | 2 |
| Bronchial Inflammation .. .. | 1 | 0 | 1 |
| Chronic Bowel Complaint .. .. | 0 | 2 | 2 |
| Consumption .. .. .. .. | 1 | 0 | 1 |
| Exhaustion .. .. .. .. | 1 | 1 | 2 |
| Scarlet Fever .. .. .. .. | 0 | 1 | 1 |
| Water in the Chest .. .. .. | 0 | 1 | 1 |
| Total .. .. .. .. | 8 | 7 | 15 |

*Coercion.*—"In connexion with other improvements in the treatment of the Insane, we, at an early period of our Institution, made it our especial study to render the means of coercion, when necessary, as gentle and as little irritating as possible; but some degree of personal restraint is in many cases indispensable, and howsoever gratifying the idea may be to the speculative philanthropist, the entire abolition of coercion is too often compensated by concealed severity. When we hear a vulgar and uneducated keeper boasting that, by a glance of his eye, or the turn of his finger, he can control a whole ward of the insane, we can guess pretty well how this seemingly mysterious power was acquired; and it would be well if those who visit madhouses would carefully study the countenances of the lunatics when the keeper approaches, and when he turns his back to the patient. An attentive observer might thus sometimes discover a strong and instructive contrast between the subdued and counterfeited expression in the one case, and the suspicious and revengeful scowl in the other."

There is much good sense in this. We observe that at some of our public institutions a great parade is made of the abolition of coercion. The gratifying fact is advertised in all the papers, and the public press rings with the humanity and skill of the doctors. But experience is yet wanting to confirm both, and to prove that a dangerous extreme has not been plunged into. Every body acquainted with the insane, is aware of the pernicious self-pollutions which too many are addicted to, and the injury both to body and mind which *they* must of necessity give rise to. We should pause, therefore, before we give our unqualified assent to the total, or nearly total, abolition of restraint, and wait for time to set its seal on the discretion of those who counsel it.

*Divine Worship.*—"In our last, as well as in numerous former Reports, we bore testimony to the goods effects of Divine Worship on the minds of the insane, and our further experience continues to warrant our former sentiments. In many instance, the personal and private, as well as public, ministrations of our worthy Chaplain have carried comfort and consolation to the minds of many of our patients, who are occasionally much troubled with distressing apprehensions on religious subjects. We subjoin, in his own words, a statement of the success which continues to attend his labours in the pulpit, and of the manner in which these labours are conducted:—'The exercises of Divine Worship, in which many of our patients have the privilege of engaging, are attended with the most beneficial results. This is evident from the deep interest they take in the discourses delivered to them, and the fact that these are the means of withdrawing their attention, for a short time, from their prevailing illusions. The sermons delivered in the chapel are adapted, as much as possible, to the peculiar circumstances of the audience. Everything that is conceived to have a tendency to agitate their minds is carefully avoided, and pains taken to present them with the most soothing and practical views of Divine truth. Two very important advantages, then, are derived to our patients from the institution of Public Worship;—it is of use in alleviating the malady under which they labour, and in strengthening and gratifying those pious feelings, from which (in their secluded state) they derive the greatest consolation.'"

II. It is stated in the second Report before us that:—the patients admitted, cured, and relieved, exceeded in number those of any former year since the opening of the Institution, while the proportion of deaths has been below the usual average.

Of the patients dismissed in the course of the year, the cured amount to 47.368 per cent.; the relieved to 27.067 per cent.; those removed before they had received the advantage of a full course of treatment, to 7.518 per cent.; improper cases, having, after due trial, been found to be harmless and incurable, to 6.766 per cent.; and the deaths to 11.278 per cent.

Among the cures, two were remarkable for the duration and obstinacy of the disease. One of these patients, at the date of his dismission, was fifty-five years of age, and had been under treatment for nearly ten years. Latterly, the paroxysms became less frequent and less violent; and before he was dismissed, he was subjected to a probation of nearly twelve months. No symptom of his malady having betrayed itself during this period, while, at the same time, he worked willingly and steadily at his trade, he was dismissed cured; and having for some time maintained himself and his family by his industry, he has since been engaged as one of the tradesmen of the establishment, and is now a steady, useful servant, and capable of taking charge of those patients who follow his own trade. The other, at the date of his dismission, was fifty-seven years of age. He had been insane for sixteen years, during only eight of which, however, he had been subjected to medical and moral treatment. In this case also, the patient underwent probation for nearly a year, during which time he betrayed no symptom of his former malady, and worked most diligently and cheerfully in the garden. When the period of dismission arrived, he left the house, of which he had been eight years an inmate, with the greatest reluctance, and begged most earnestly to be allowed to remain as a servant. From various circumstances, we found ourselves constrained at that period to refuse his request; but having maintained himself for about a month by his industry, and there being an opportunity of employing him in the house and grounds, he was, to his great joy, hired at a small rate of wages by the Superintendent.

These cases are highly encouraging to the friends of the insane, and tend strongly to show the erroneous nature of an opinion still entertained by some, that when the disease has lasted a certain time, it necessarily becomes incurable.

*Cataleptic Ecstacy.*—There have been no fewer than five cases of this kind in the Institution.

"In three of these, the disease was complicated with a mild variety of mania, accompanied with highly excited religious feelings. One case was peculiarly interesting. No matter how the patient was occupied when the fit came on, he suddenly assumed a statue-like posture, with the trunk slightly bent, one arm elevated, and the fore-finger steadily pointed, as if at an object; the eyelids opened to their utmost extent; the eyes turned up; the lips separated, and the expression of the whole countenance that of the most profound admiration, wonder or rapture. In this state the patient would remain a considerable time, totally unconscious of what was passing around. The whole appearance of these patients betrayed an intense concentration of thought on some hallucination, though we never could obtain any satisfactory explanation of the state of their minds while thus affected. In none of the cases during the fit, was there any unusual paleness, or flushing, or irregularity of the circulation, as described by some authors; and they could generally be easily roused by addressing them in a loud voice, or suddenly taking hold of them. Sudden noises, such as the falling of a chair, the slamming of a door, &c. made no impression on them. One of them, during his fits, was in the habit of repeating a series of doggerel verses, five or six consecutive lines rhyming together, and the matter consisting of exclamations, rapid questions, and short assertions, all expressive of intense devotional rapture. In none of these cases has the mind as yet become perfectly sane; and, in some, there is reason to fear that organic lesion of the brain has taken place."

There is one point insisted on by the Reporters which we cannot avoid alluding to. Most medical men must have deplored the infatuation of relatives and friends, and seen instances of deplorable results from their well-intentioned but injurious interference.

"After all delusions have vanished, and where there is no evident periodicity, a certain term of probation is required for the establishment of mental government, both morally and intellectually, in order to enable us, with any confidence, to pronounce the patient to be completely cured. In the course of the year, several patients were removed by their affectionate, but ill-judging relatives, notwithstanding every remonstrance, and, as we had predicted, were in a few days, one of them in a few hours, brought back to the Asylum in a state of complete frenzy. We cannot too strongly impress on the minds of the friends of the insane, that, if there be a return of a single delusion, moral perversion, or even sleepless night, so far from being cured, the patient is not even convalescent, but in imminent danger of a return of a paroxysm of his malady. We must no less strongly condemn what we have found to be a very common error, that of removing the patient, only for a short time, *by way of trial*, with a view to bring him back, if necessary, to complete his cure. We have, in many cases, both now and in former years, had to regret the interruption thus given to the proper treatment of the patient, leading generally to protracted, and not unfrequently, to incurable insanity."

*Diet.*—The following observations are interesting.

"The proper regulation of the diet of the insane, is a point of great importance. In most cases, especially in those of the poorer classes, who are often, when admitted, much debilitated and emaciated, we have found benefit from a diet rather generous and restorative; but we are well aware that even where there is no evident tendency to inordinate vascular action in the system, a degree of feverishness commonly succeeds a full meal. The pulse becomes accelerated, the face flushed, and the general heat of the body increased. Such effects of food are particularly remarkable in cases of lunacy, in which these and other well-known symptoms often indicate an increased determination of blood to the head. The frequent repetition of this state, tends to produce, or to exasperate a paroxysm of lunacy; and, ultimately, to terminate in apoplexy, or other fatal condition of the brain. While, therefore, we have recently adopted some changes in the rotation of our articles of diet, introducing (especially among patients of superior rank) a more gratifying variety of food, yet this is always done with careful discrimination. In general, where there is great excitability, the food must be in moderate quantity, and of the least stimulating quality. We have had many instances of patients who became quite unmanageable, if indulged with the smallest daily allowance of animal food, but who remained perfectly quiet and submissive, so long as they were confined to vegetable fare. In some cases, however, the pulse flags, the flesh wastes, and the strength fails, even while the maniacal paroxysms still run high; and we can neither reduce by evacuation, nor interfere with the appetite, by the use of those narcotic and nauseating drugs, which in other cases might safely and usefully be administered. In such cases, we have recourse to the abstraction of some of those other stimuli which often serve, in no small degree, to increase the excitation of the insane. In the highly excitable lunatic, the stimulating influence of light has long been well known; but the salutary effect of the abstraction of that stimulus, although only during the more active process of digestion, has, we believe, not been hitherto noticed. In several cases of the description alluded to, we have found, that if immediately after dinner, the patient be placed in a chamber from which all light has been carefully excluded, and thus kept in utter darkness for about an hour or two, the excitation occasioned by the stimulating effect of food, is greatly diminished, if not entirely subdued. The daily use of this very safe and simple means of treatment, has often had a durably beneficial effect, especially when aided by the influence of quietness, solitude, cooling applications to the head, and warmth to the lower extremities.

In some cases of lunacy the appetite is voracious, while in others, it is no less remarkably defective; and in the course of the year, no fewer than six of our patients utterly refused food. It is a point of no small importance, to decide whether the refusal of food be the consequence of delusion, or of the state of the digestive organs: but an attentive observer will generally have no great difficulty on this point, while a single trial of the operation of feeding will usually be decisive. In the case of one of the six patients alluded to, it was presently evident, that the patient was fast sinking, and therefore, unless for the purpose of occasionally conveying a little wine or other cordial into the stomach, any compulsory feeding would have been but of little avail. All our other cases were treated with our usual success. In several of them the patients resumed the use of their powers of deglutition after only one or two feedings. In one of these cases, however, we had occasion to feed for two months; in another, for the still longer period of three months; and thus not only sustaining the strength of the patients, but with evident increase of bulk and weight, and with even a tendency to corpulency. Suicide, by means of starvation, was the object in both of these cases; and of all the motives for abstinence, this is perhaps the most obstinate. The food given consists of nutritious fluid, injected into the stomach by a peculiarly simple and effectual apparatus.

One of these patients had for some time been observed to be very attentive in keeping the food comfortably warm at the fire, before the operation of feeding commenced, and to express no small degree of anxiety for the arrival of the operator. Taking advantage of this circumstance, one day immediately before the time of feeding, the patient was informed that an accident had befallen the apparatus, which unfortunately rendered it useless, and, as it was Sunday, when it could not be repaired, the feeding must necessarily be postponed till the following day. On hearing this very mortifying communication, the patient instantly seized the vessel containing the fluid, and presently swallowed the contents with avidity, continuing thenceforward to take food, whether solid or fluid, without reluctance. We have now had not a few years' experience of the use of our feeding apparatus, and while we feel gratified to learn that it has lately been adopted in other establishments, we are confident that it needs only a trial to evince its superiority over every other mode of compulsory feeding hitherto employed."

*Occupation.*—The Report is, on this head, as satisfactory as ever. But we have dwelt on the subject so frequently, that we do not think it necessary to advert to it further at present.

*Intemperance.*—The Reporters bear testimony to its deadly operations in leading both to insanity and death.

*Mortality.*—The number of the patients who died, and the denomination of the diseases which proved fatal, were as follows:—

| | *Males.* | *Females.* | *Total.* |
|---|---|---|---|
| Of Abscess .. .. .. .. .. | 1 | 0 | 1 |

| | *Males.* | *Females.* | *Total.* |
|---|---|---|---|
| Apoplexy or its consequences .. .. | 3 | 0 | 3 |
| Chronic Bronchitis .. .. .. | 0 | 1 | 1 |
| Consumption .. .. .. .. | 0 | 3 | 3 |
| Contusions previous to Admission .. | 0 | 1 | 1 |
| Diarrhœa .. .. .. .. | 1 | 1 | 2 |
| Diseased Urinary Organs .. .. | 1 | 0 | 1 |
| Erysipelas .. .. .. .. | 1 | 0 | 1 |
| Exhaustion .. .. .. .. | 1 | 1 | 2 |
| Total .. .. .. .. | 8 | 7 | 15 |

These Reports are very clearly and satisfactorily drawn up.

---

### THE TWENTY-FIRST REPORT OF THE DIRECTOR OF THE WEST-RIDING OF YORK PAUPER LUNATIC ASYLUM.

FROM this brief Report, we extract the following passage bearing on two points which occupy the attention of medical men at present—the classification of the insane and coercion.

The patients are classified according to the divisions,—Mania, Monomania, Melancholia, Dementia, and Idiotcy. The Maniacs and Idiots are kept entirely apart from the rest, in a distinct part of the building. The Melancholics are not permitted to associate together, but are surrounded by those most likely to divert and cheer them; and when the weather will permit, are employed, the greatest portion of the day, in various occupations out of doors.

The instances of restraint are few; of the 201 male patients now in the house, only one is under confinement, three maniacal females are restrained, and two less violent partially so.

No one is placed under restraint, but by the immediate order of the Director, and as soon as the symptoms warrant their liberation, all restraint is removed; many instances have, however, been known of patients who, feeling a return of excitement, have themselves requested to be again restrained; a proof that when they could exercise a judgment, they were sensible how beneficial it had been to them.

To permit patients in a high state of excitement to keep up that excitement by constant muscular action, or knowingly to risk the lives of both patients and servants, would be treatment having no more of humanity in it than the name; and it requires but little practical acquaintance with the subject at once to detect its absurdity.

The result of many years' careful attention to this subject, has led to the conviction that a mild and judicious restraint can never be supplied by any "surveillance." The presence of any individual is of itself sufficient, in many instances, to keep up the excitement; for it is a truth but too general that maniacs regard all around them as enemies, and exhaust themselves in vociferation, and attempts at violence; whilst force on the one part, and resistance on the other, keep up the unequal contest, ending sometimes in bruises or broken limbs.

No servant is permitted to strike a patient under the penalty of losing his situation, nor is any harsh language allowed on their part, but a continued exercise of forbearance and uniform good-will is required from them; it is, therefore, of vast importance to retain experienced servants in the charge of the insane. A change can seldom be made in the attendants without disadvantage to the patients; strangers are more easily irritated by the offensive language, and sooner intimidated by the threats and violence of those entrusted to them and, on the other hand, the insane are especially acute in discovering the exact capabilities of their attendants, and in making use of such discovery to their own, no less than to the disadvantage of others.

---

### An American's Account of the Lunatic Asyla in Europe.*

Dr. Pliny Earle, during a recent tour in Great Britain and on the Continent of Europe, visited thirteen Asyla for the Insane, and on his return to America, published, in our contemporary of Philadelphia, a brief sketch, or rather reminiscence of what he had observed. Nations, like individuals, do not know themselves. They should listen to what strangers say of them. We shall not follow Dr. Earle into every madhouse, nor transcribe every remark. We shall merely pick out what may serve to instruct or to amuse.

### *County Lunatic Asylum at Hanwell.*

The most prominent characteristic in the internal economy of this institution, is the amount of labour performed by the inmates. Perhaps no other Asylum of the kind can furnish so great a per centage of patients devoted to useful occupations. Of the *six hundred* who were there in 1837, more than *four hundred* were thus employed, most of these were incurables.

### *The Pauper Lunatic Asylum for the West-Riding of York at Wakefield.*

The number of patients in the Asylum during the Summer of 1837, was 334, of whom a small minority were women. Fifty or sixty of the men labour, regularly, either in the manufacture of the articles above mentioned, in gardening, or in some mechanical trade.

"Nearly two hundred dollars per annum is paid for tobacco, which is also divided among the labourers, each being entitled to a weekly ration of one ounce. Many of the patients, as we passed through the wards, begged us for tobacco, or for money to purchase it with. One of them, after having thus played the mendicant, put into our hands a piece of cloth, upon one side of which he had written, in large letters, '*Millennium. Green, blue, and yellow united.*' And upon the other, '*Victoria* 1*st, July* 28*th*, 1837. *Virgin Queen of Peace. Amen. Aquila.*' It will be perceived from the date, that this was but a short time subsequent to the accession of Victoria to the throne of Great Britain. The universal popularity which the youthful queen enjoyed, at that time, among her sane subjects, seems to have been participated also by some of those who were insane. And this poor infatuated maniac beheld the 'green, blue and yellow,' the insignia of the different political parties of the realm, united through her means, and hence the consummation devoutly to be wished, the immediate advent of the millennium! 'Eh, eh,' said he, after I had read the above, and, as he spoke, he looked up into my face with a piercing glance and a most significant smile, 'do you know what Aquila signifies in English?' Being answered in the affirmative, 'Well, Sir,' he continued, '*I* am the Eagle.'

The number of patients admitted to treatment in this Asylum, from the time it commenced operation, Nov. 23d, 1818, to January 1st, 1837, was 2,224, viz.

| | Males. | Females. | Total. |
|---|---|---|---|
| | 1,150 | 1,092 | 2,242 |
| Of whom there have died | 420 | 289 | 709 |
| " " " been discharged | 560 | 664 | 1,224 |
| " " " are remaining | 170 | 139 | 309 |

---

* American Journal of the Medical Sciences, Nov. 1839.

The total number of cures was 991, equivalent to $44\frac{1}{2}$ per cent. The number relieved, but not cured, was 233. The deaths are equivalent to $31\frac{6}{10}$ per cent.

Dr. Earle gives a short table illustrative of the benefits of treatment during the acute stage of insanity, and of the disadvantages of delay.

| | Admitted. | Cured. | Died. | Discharged not cured. | Remaining. |
|---|---|---|---|---|---|
| 1st. Within 3 months of the First attack, | 682 | 382 | 163 | | |
| 2nd. " 12 " " | 409 | 212 | 149 | | |
| 3d. Between 1 and 30 years from " | 516 | 58 | 265 | | |
| 4th. Those who had previously been insane and confined in this Asylum, | 288 | 339 | 132 | 233 | 309 |
| 5th. Those who had been previously insane but not treated in this Asylum, | 347 | | | | |
| Total .. .. .. .. | 2242 | 991 | 709 | 233 | 309 |

Of the 1st division there were cured, 56.01 per cent., and 22.9 per cent. died; of the 3d division 51.83 per cent. were cured, and 36.43 per cent died; of the third, 11.2 per cent. were cured, and 51.35 per cent. died; of the 4th and 5th included together, 53.38 per cent. were cured, and 20.79 per cent. died.

*The Retreat near York.*—Dr. Earle speaks of this in high terms. After describing the building he says,—The classification of the insane is founded, principally, upon their ability or willingness to pay for the accommodations afforded. There are four classes, in the lowest of which the price is fixed at *four* shillings, sterling, per week, while in the highest it varies from about *twenty* to *eighty* shillings. Those who pay the price last mentioned have two rooms, elegantly furnished, and a special attendant. System and neatness prevail in every department, and elegance is added to that of the class last mentioned. He adds, in England, as in the United States, the officers of the Lunatic Asylums complain of negligence on the part of the friends of insane persons, in omitting to place them under their care until the disorder has assumed a chronic character, and, consequently, the probability of a cure exceedingly diminished. In order, if possible, to remedy this evil, fraught, as it is, with consequences of so fearful a nature to the unfortunate sufferers, the directors of the Retreat have ordered that an abatement of four shillings per week during the first year of the patient's residence in the Asylum, be made from the expenses of those who enter within six months of the first decisive symptoms of the dreadful malady.

It appears that, up to 1837, the cases in this celebrated establishment were as follows:—Of the patients seen during the first attack and admitted within three months of its commencement, are equal to 79.7 per cent.; of those between 3 and 12 months, 43.9 per cent.; of those of the second attack, and whose disease was less than 12 months' duration, 59.4 per cent.; and of those of more than 12 months, 25.8 per cent. The cures of the whole equal 46.5 per cent. and the deaths 22.2 per cent.

*York Lunatic Asylum.*—The number of insane in the Asylum, in the summer of 1837, was 170. There are accommodations for 200. The patients are divided, as in the Retreat, into four classes, the principle forming the basis of classification being the same as at that asylum. Among the amusements permitted are cards and billiards.

The number of patients admitted to this Asylum from the time of its going into operation, in November, 1777, to October 10th, 1814, was 2635. Of these there were discharged, either cured, improved, or at the request of friends and guardians, 2133. Of the remainder, 399 died, and 103 remained in the Asylum at the latter date. The deaths during this period were equal to 16.8 per cent. Again, from October 10th, 1814, to June 1st, 1837, a period of 22 years, 4½ months, there were 1131 admissions, which, together with the 103 remaining at the former date, makes a total of 1234. Of these there were 387 cured, 224 improved, 247 removed by their friends, and 217 deceased. There were remaining in the Asylum, at the date last mentioned, 83 men and 76 women, a total of 159. Excluding this 159, as being still under treatment, we have for the cures 36 per cent. and for the deaths 20 per cent.

At this institution, any servant who strikes or otherwise mal-treats a patient is dismissed. If a patient escape, the expense of retaking him is defrayed, either wholly or in part, at the discretion of the committee, by the servant having charge of him.

Let us turn to the Continent, and contrast their mad-houses with our own.

*Asylum at Amsterdam.*—The building is of somewhat antique construction. Each ward, like those of most hospitals for the sick, is without subdividing partitions, the beds being arranged upon either side, and, in this instance, rather too compactly. The wards are, moreover, like those in the hospitals for the sick in Amsterdam, in that they are two stories in height, a platform, or gallery, running around, above the beds, between the first and the second story. This is used as a place of promenade for the patients. There are seven wards, four for women and three for men. The courts devoted to the use of the patients, and of which there is but one for each sex, are very small, and being without shrubbery, flowers, or even green-sward, have the naked and forbidding aspect of a prison-yard. Natives of the city of Amsterdam alone, are admitted into this Asylum. The patients are mostly paupers, or subjects of charity. There are six beds, in small, decently furnished rooms, which are intended for pay patients. The number of patients, in July, 1838, was 157. Of these 69 were men, and 88 were women. A large majority of them were incurable. " During the two years and eight months ending in July, 1838, there had been admitted 85 men and 78 women, or a total of 163. In the same period, 27 men and 29 women had been cured, and 40 men and 37 women had died. As means of coercion and punishment the hands and feet of patients are sometimes fastened, and the camisole, the straight-jacket and imprisonment are resorted to. For the last mentioned purpose there are six dungeons, constructed three upon either side of a small apartment. One of these was occupied, at the time of my visit, by a woman, who was naked, raving and filthy."

A pretty place this Amsterdam Madhouse. A radical reform is promised—not before it is wanted! There is in it neither labour nor amusement. " A few of the women were either knitting or sewing, but the men, without exception, were unoccupied, lying on the floor, the ground, or the beds, standing in the stupidity of dementia and idiocy, or walking to and fro, raving with the unbridled fury of madmen." No wonder! This is just the place to make a man mad.

*Asylum at Utrecht.*—Dr. Earle speaks favourably of this. " The bedsteads," he says, " for the most maniacal patients, and such as are not the most cleanly, are somewhat different from any others which I recollect to have seen. They are made of boards, in the form of a child's crib, though deeper, and the bottom is concave or descends in every direction to the centre, where there is an aperture for the escape of water. There is a common sitting-room for each class of the inmates. The number of patients, at the date first mentioned, was 94, that of the two sexes being about equal. They are divided into three classes, the basis of division being the sum paid for entertainment."

The patients resort to reading, writing, drawing, music, cards, billiards, chequers or draughts, and some other games, for amusement. There is a library intended for their use. The billiard-table, a large and handsome one, was made by two of the former patients. In one of the men's rooms several patients were occupied in drawing and reading. Yet the Asylum of Utrecht still retained some traces of the barbarous olden time, for, when necessary, the camisole or the straight-jacket, fetters, the douche and the dungeon, are put in requisition as means of punishment. The stream of water forming the douche is but one-fourth of inch in diameter, while those of Salpêtrière and Bicêtre, at Paris, are about seven-eighths of an inch. The quantity of water flowing from the latter must consequently be nearly twelve times as great as from the former. There is but one bathing-tub belonging to the establishment, but the accommodations in this respect are about to be increased.

*Asylum at Antwerp.*—There is not much to be admired in this. The building, composed of brick, is very old. It is but two stories in height and encloses several small courts, which " blossom as the rose." The doors of the dormitories are still fastened with huge bolts at top and bottom. The rooms referred to are arranged on both sides of very narrow passages leading through each ward. There are 24 cells for the raving maniacs, 12 for those of each sex. These are small; the wainscoting is of wood, in order to diminish the danger of the patients injuring themselves against the walls; and each contains no other furniture than a bed. These beds are low, made of plank, and fastened to the walls. The mattrasses, throughout the building, are mostly of straw; those of the convalescent and of the pay patients are, however, of better materials. In the infirmary the beds are very good. A few years since the proportion of incurables was very large; but the Grippe, which prevailed so generally, in an epidemic form, throughout the west of Europe, during the winter of 1836-7, carried off many of them, and, subsequently, most of those who had been attacked by it and recovered, became victims to phthisis pulmonalis. Each class of patients has a court and a common hall, in which they spend most of their time. The halls are warmed by stoves, which are surrounded, at a few feet distant, by a strong reticulated wire fender. The dormitories, or private rooms of the patients, as well as the cells for the furious, are not furnished with the means of being warmed. This is to be remedied. Both labour and amusement are very imperfectly employed.

*La Salpêtrière.*—This vast asylum for the poor, this pauper-village, if the term be admissible, was established by Louis XIV. in the year 1656. It is devoted exclusively to females, for whom it contains about 4500 beds. It is in the hospital of this extensive establishment that the celebrated Cruveilhier has collected most of the materials for his elaborate and beautiful works upon pathological anatomy. The department devoted to the insane is, perhaps, the most extensive in the world, the number of patients usually exceeding 1000. The approximate number of admissions, per annum, is 500, that of discharges 300, and of deaths 200. The cures are equal to 33⅓ per cent. of the whole number admitted. Though Pinel here introduced those ameliorations in their treatment which should induce madmen to raise a monument to his memory, the Institution does not appear to be quite *au niveau* of the best. They are now, at Esquirol's suggestion, making all their new buildings but one story in height.

*Le Bicêtre.*—The " hospice," or pauper Asylum of Bicêtre, is in a southerly direction from Paris, about two miles from the walls at the barrière de Fontainbleau. It is for men alone. Its extent is less than that of La Salpêtrière, the number of inmates being about 3000. The department for the insane is proportionally smaller. The number of patients in May, 1838, was 760. The average number of admissions, annually, is, for the insane, 360 ; the imbecile 40 ; that of discharges of the former, per month, from 12 to 15 ; the deaths about the same, and the cures from 7 to 8. The proportion of deaths to the whole number admitted is as 1 to 6, or 16.66 per cent. The medical care of the patients is confided to Drs. Ferrus and Pinel.

The douche is employed here, and we should suppose, from Dr. Earle's account of it, with little mercy. It gives us no great opinion of Dr. Pinel's practice to find him trying, literally, to drive a notion out of a madman's head, by means of it. We have not room for the scene, which is painted humourously enough by Dr. Earle.

*Asylum at Charenton.*—The Médecin en Chef here is the venerable Esquirol. It includes many edifices, which have been erected at various periods, and extensive gardens and promenades, which extend to the summit of the hill upon the declivity of which it is located. The section for men is composed of four courts, of which three are planted ; three infirmaries ; one ward (salle) for patients of a suicidal propensity ; one dormitory ; one gallery and six corridors, into which open the doors of the several rooms ; one bathing room, and six rooms where the patients assemble. These last mentioned can be heated. The section for women has a garden, four planted courts, two infirmaries, one ward for women disposed to commit suicide, two bathing rooms, seven dormitories, six galleries and corridors into which open the doors of the apartments, and five rooms in common, which may be heated. An extensive additional department for females, combining most of the modern improvements, was erected about ten years since, and first occupied in 1829. Dr. Earle speaks very highly of *it*. There is a parlour in the Asylum, in which those patients the state of whose disease renders them admissible, assemble every evening for social intercourse. This contains many arm-chairs, several card-tables, and a piano-forte. A room having tables for billiards, is devoted to that amusement. The bathing room of the new department for females contains ten copper tubs, separated from each other by curtains, and each supplied with a cover which may be used in case of necessity. The number of *attachés* to the Asylum is 170.

The number of admissions, from the establishment of the institution exclusively for lunatics, in 1797, to the end of the year 1833, is 5972. The following list exhibits the same number divided in the proportion that the patients were received in several different epochs.

| | |
|---|---|
| From 1797 to 1802 ........ | 202 |
| 1802 " 1805 ........ | 435 |
| 1805 " 1817 ........ | 1007 |
| 1810 " 1815 ........ | 722 |
| 1815 " 1825 ........ | 2049 |
| 1825 " 1834 ........ | 1557 |
| Total, ........ | 5972 |

Previously to 1815 the number of each sex was not designated, but from that year to 1825 there were 1245 men and 804 women ; and from 1825 to 1834, 932 men and 625 women.

Dr. Earle enters into a number of statistical calculations, for which we have not space, and which, in fact, are mainly taken from Esquirol's book. The average number of cures, per annum, was, for men, 30.32 per cent ; for women, 39.13 per cent ; and for both sexes, inclusive, 34.72 per cent. The average of deaths was, for men, 43.61 per cent ; for women, 22.07 per cent ; and for both, 32.84 per cent.

A variety of circumstances on which it is unnecessary to enter will account in some degree for the high ratio of mortality.

*Asylum at Milan.*—Saint John's Hospital, at Milan, is, as is well known, one of the most extensive institutions of the kind in the world. It is probably exceeded by none. The principal edifice appertaining to it is within the city, but the department for the insane is without the walls, about a mile distant from the Porta Tosa. It encloses large courts, but those which are devoted to the use of the patients are chiefly external to it. The wards are very large, some of them consisting of a hall, of ample width, running the whole length, and, beside it, two or three dormitories, each containing numerous beds. In others there are dormitories upon both sides of a narrow passage. The rooms are all lofty and well ventilated. In those where a fire is required, the stove is placed in the middle of the room and covered on all sides with bricks and surrounded by strong wooden frames or fenders. The beds for the furious have confining rings and straps at the foot and the sides, like those in many other Asylums. The mattrasses are made, some of straw, some of wool, and some of hair; and the beds and linen are as neat, though not so elegant, as those in any institution, Dr. Earle has visited. In some of the dormitories metallic *pôts-de-chambre* are fastened by straps to the bedsteads. In the third story of the department for women there is a ward consisting of a suite of small rooms, each opening from a narrow passage. The bed in each of these is beneath the window, at the extremity opposite the door. Beside it, in the corner, is a stool of convenience, communicating with a balcony on the outside of the building, where a servant can attend to it without entering the room.

One ward in the department of each sex, in the Asylum under notice, is devoted to convalescent patients. In these they remain forty days, previously to leaving the Asylum, for the purpose of confirming a cure. A horizontal jet, in which the water is thrown, with considerable force, completely across the room, is intended for a similar purpose as the douche. Being thus arranged it may be more easily applied to the various parts of the body.

The number of patients, in Nov. 1838, was 420, of whom 215 were men and 205 women.

Manual *labour* is pursued to a considerable extent both by men and women. The only means of amusement are a swing and a "giustra." The latter is so constructed that four, or, indeed, eight persons may turn horizontally in a circle, being situated at the extremities of two beams which cross each other at right angles, in the centre. These are in the principal court occupied by the men. The court is shaded by rows of sycamore trees, with seats fastened to the ground.

*Asylum at Venice.*—A large edifice belonging to the civil hospital. It is three stories in height, and entirely surrounds a court about 100 feet square. A corridor passes around this court, furnishing shelter from both sun and rain. In November, 1838, this Asylum contained 230 patients, all of whom were women. Upon the island of San Cervilio there is another establishment, for the men, of whom the number varies but little from that of the women. The patients at the Asylum for the women are divided into six classes, according to the species of insanity. These classes are not kept exclusively separate; on the contrary, some individuals belonging to every one of them may be found in the same ward. The colour of a strip of cloth attached, as an epaulette, to the shoulder of each patient, is a mark by which those belonging to the several classes may be identified. The classes are, 1st, *mania*, distinguished by a *red color;* 2d, *monomania*, distinguished by *deep blue;* 3d, *melanconico*, by *green;* 4th, *idiotismo*, by *orange;* 5th, *stupidita*, by *light blue;* 6th, *demenga*, by *yellow*.

The *third story* is a suite of three rooms, called *Sicurezza*. They contain about 50 beds, all of which were occupied. The bedsteads are made of plank, similar in form to those at the Asylum at Utrecht, but differing from them, in that the bottom is a rack, thus permitting water to escape in all parts. A large box or trough is placed beneath each, to preserve the cleanliness of the floor. The patients in this department mostly lie upon loose straw, which is covered by a blanket. Many of them were confined by having their arms fastened to the sides and their feet to the foot of the bedsteads. This does not say much for Venice!

*Asylum at Malta.*—About 1 in 900 of the population of Malta is mad. The asylum is at Floriana, in the suburbs of Valetta—an old and incommodious building, containing about 90 patients, 40 men and 50 women.

Manual labour has been introduced—not so amusement, nor even reading and writing! "Whenever," says Dr. Earle, "bodily restraint is necessary, the camisole or straight-jacket is called in requisition. The pattern of this garment of coercion, used in this Asylum, is, in my opinion, an improvement upon all others which have come under my observation. Its principal peculiarity consists in two bands, one upon either side, attached to its lower border and passing around the legs. The whole garment is thus kept more effectually in its proper place. The patients have ever been, and still are, mingled together, irrespective of stage or intensity of disease." We cannot say much for our island of Malta.

*Asylum at Constantinople.*—A hospital adjacent to the mosque of Suliman is devoted exclusively to the insane. There, none but men are admitted; the women, according to the Turkish custom, as well as in conformity with the precepts of the religion of Mahomet, being kept in private seclusion. The building is but one story in height, and, like the cloisters of many Gothic cathedrals, and the khans or caravanseras of Turkey and Natolia, completely surrounds a central court. With our author's account of what he saw here we conclude.

"We passed along the corridor to the first window. From between the bars of the iron grating with which this was defended, a heavy chain, ominous of the sad reality within, protruded, and was fastened to the external surface of the wall. It was about six feet in length, the opposite extremity was attached to a heavy iron ring, surrounding the neck of a patient who was sitting, within the grating, upon the window seat. We entered the room and found two other patients, similarly fastened, at the two windows upon the opposite side of the room. It was a most cheerless apartment. A jug to contain water, and, for each of the patients, a few boards, laid upon the floor, or elevated three or four inches, at most, and covered with a couple of blankets, were all the articles of comfort or convenience with which, aside from their clothing, these miserable creatures were supplied. Although in the latter part of December, they had no fire; nor were the windows glazed, but close shutters attached to each, rendered it possible measurably to shield the inmates from severe weather whenever it might occur. The length of the chain of each patient is barely sufficient to enable him to lie down upon his comfortless bed of boards and blankets. Leaving this apartment, we proceeded successively to the others, twelve or fifteen in number, in all of which we found the patients in a very similar condition to those whom we had first seen. There was but one who was not chained. He was an elderly man, though still retaining much of the vivacity of earlier years. His long and profuse hair and beard were nearly white, and his complexion very delicate. He was formerly a priest of the Islam faith. He has been deranged and confined in this place nearly fifteen years, during which time he has thrice broken the chain with which he was secured. He is now alone in his apartment, within which no one is permitted to enter. He talked and raved incessantly, threatening to kill those who were making him their gazing stock.

Like those in the apartment first mentioned, all the patients, with one exception, were without fire. The person forming this exception was one of the most hideous of undeformed human beings. He has been in the Timar-hané, as this Asylum is called by the Turks, more than forty years. His hair and beard, both naturally abundant, curly, and black as ebony, appeared as if they had not been cut or combed since his entrance. They nearly concealed his face, and the former hung in a profusion of literally 'dishevelled locks' about his neck and shoulders. His head would have been a *nonpareil* for an original to the figure of Cain, in David's celebrated picture of 'Cain meditating the death of Abel.' He lay crouched upon all-fours, resting upon his knees and elbows, and holding his head and hands over a *manghale* of living embers."

The other patients appear to enjoy pretty good health. A physician attends them regularly. There is a peraon who has the charge of supplying them with food, and they receive considerable attention from those who visit them, and give them articles of food, money, tobacco, and fill and light their *chebouks*.

